# The inverted U-shaped relationship between weight loss percentage and cardiovascular health scores

**DOI:** 10.1007/s40519-023-01619-3

**Published:** 2023-10-24

**Authors:** Feng Chen, Yu Zhang, Shaohe Chen

**Affiliations:** Department of Child Healthcare, Wenzhou People’s Hospital, Wenzhou, 325000 China

**Keywords:** **O**besity, Weight loss, Cardiovascular health, National Health and Nutrition Examination Survey

## Abstract

**Purpose:**

Obesity is a significant risk factor for cardiovascular disease; however, the impact of weight loss on cardiovascular health (CVH) in individuals with specific obesity patterns remains incompletely understood. The objective of our study was to investigate the relationship weight loss percentage and CVH scores across individuals with various obesity patterns.

**Methods:**

Our study utilized data from the National Health and Nutrition Examination Survey conducted between 2007 and 2018, involving a total of 12,835 participants aged 16 years or older, to conduct a cross-sectional analysis. Multiple linear regression and multinomial logistic regression methods were used to assess the correlation between the weight loss percentage and the CVH scores. Additionally, restricted cubic spline analysis was employed to examine the nonlinear relationship between the two variables.

**Results:**

Compared to individuals with a weight loss percentage < 0%, participants with weight loss percentages of 0–5% and 5.1–10% showed improved CVH scores, with β values of 2.85 (95% CI 2.32–3.38) and 2.55 (95% CI 1.69–3.4), respectively. Regarding different obesity patterns, compared to participants with a weight loss percentage < 0%, participants with a weight loss percentage of 0–5% showed an increase in CVH scores in the normal weight and overweight/general obesity (OGO) groups, with β values of 1.45 (95% CI 0.7–2.19) and 1.22 (95% CI 0.46–1.97), respectively. Restricted cubic spline analysis revealed a significant inverted U-shaped relationship between the weight loss percentage and the CVH scores (with optimal CVH scores at 3%).

**Conclusions:**

There was an inverted U-shaped relationship between weight loss percentage and CVH scores, with moderate weight loss (0–10%, optimal value of 3%) being associated with improved CVH scores, especially among individuals with OGO.

**Level V:**

Opinions of respected authorities, based on descriptive studies, narrative reviews, clinical experience, or reports of expert committees.

**Supplementary Information:**

The online version contains supplementary material available at 10.1007/s40519-023-01619-3.

## Introduction

Obesity and its association with cardiovascular diseases (CVDs) present a considerable challenge in the field of global public health [1–6]. In 2015, approximately 604 million adults worldwide were affected by obesity [7]. Moreover, CVD is one of the leading causes of mortality globally, resulting in approximately 18 million deaths each year [8]. Obesity has long been recognized as a crucial risk factor for CVD and is closely associated with various metabolic abnormalities, such as hypertension, dyslipidemia, hyperglycemia, inflammation, and endothelial dysfunction [9–13]. These factors contribute to the accelerated development and progression of atherosclerosis. Moreover, obesity impacts cardiac structure and function, leading to complications such as myocardial hypertrophy, dilated cardiac chambers, heart failure, and arrhythmias [14]. As a modifiable risk factor for CVD, weight loss is particularly important for improving cardiovascular outcomes and reducing mortality rates associated with CVD [15–17].

In 2010, the American Heart Association (AHA) introduced the “Life’s Simple 7” (LS7) scoring system. This scoring system encompasses seven indicators, including diet, smoking, body mass index (BMI), physical activity, total cholesterol, blood pressure, and blood glucose [18]. The LS7 scoring system has been widely utilized in cardiovascular disease research as a means to assess the cardiovascular health (CVH) status of populations. Previous studies consistently found that improving cardiovascular health (CVH) indicators can reduce the incidence of CVD complications and mortality rates [19–25]. In 2022, the AHA updated and improved this scoring system by including “sleep health” as a factor, thus allowing a more comprehensive assessment of an individual’s CVH [26]. Since the introduction of “Life’s Essential 8” (LE8), studies have confirmed the association of these scores with cardiovascular outcomes and all-cause mortality [27]. However, to our knowledge, research on the relationship between weight loss and overall CVH remains scarce. Currently, clinically significant weight loss generally refers to a reduction of 5% or more relative to baseline weight [28, 29]. A study has demonstrated that a weight loss of ≥ 5% from baseline can improve lipid levels, glycemic control, and blood pressure management; however, these benefits are mainly observed in individuals with cardiovascular risk factors [30]. Therefore, based on extensive data from the National Health and Nutrition Examination Survey (NHANES), we aimed to investigate the correlation between weight loss and CVH scores. Additionally, we compare the outcomes among individuals with different patterns of obesity to achieve a more precise evaluation of the impact of weight loss on cardiovascular health, thus providing robust evidence for clinical practice and informing the development of public health policies.

## Materials and methods

### Study population

**Fig.1:**
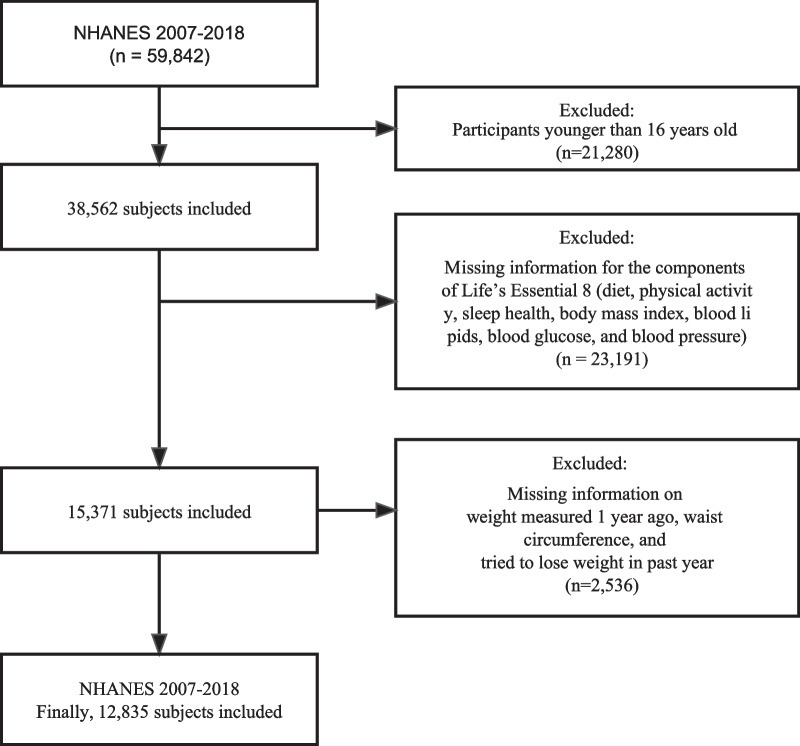
The study’s flow diagram

The NHANES is a crucial health and nutrition surveillance program in the United States overseen by the National Center for Health Statistics (NCHS). The NHANES aims to gather data on population characteristics, health status, and nutritional intake from a nationally representative sample, with the goal of supporting the development of public health policies and facilitating health research. The survey employs various data collection methods, including face-to-face interviews, physical measurements, laboratory tests, and dietary assessments. These data are released on a biennial basis, with a two-year cycle. In the present study, we collected a total of 59,842 participants using 6 continuous NHANES cycles from 2007 to 2018. Because the interviewees for certain variables (sleep duration, medication usage) were aged 16 and above, individuals younger than 16 (*n* = 21,280) were excluded from this study, as well as those with missing information pertaining to the components of the CVH scores, namely, diet, physical activity, sleep health, body mass index (BMI), blood lipids, blood glucose, and blood pressure (*n* = 23,191). Additionally, participants with missing waist circumference (WC) measurements (*n* = 169), self-reported weight from one year prior (*n* = 173), and self-reported attempts at weight loss (*n* = 2194) were omitted from the study. Ultimately, the analysis incorporated data from a total of 12,835 participants (Fig. 1). The survey procedures were authorized by the Centers for Disease Control and Prevention (CDC) and the Ethical Review Committee of the NCHS. All participants provided signed informed consent.

### Measurements and variables

In the present study, the main independent variable was the percentage of weight loss. Specifically, participants reported the difference between their weight one year ago and their current weight, which was then divided by their weight one year ago. This number was then multiplied by 100 to obtain the weight loss percentage, and participants were categorized into the following groups: weight loss percentages of less than 0% (reference group), 0–5%, 5.1–10%, 10.1–15%, 15.1–20%, and greater than 20% (Supplementary Table S3). Additionally, height and WC were extracted from the database. The NHANES website provides the detailed procedures used to collect anthropometric measurements. The selection of these covariates is based on their relevance and frequent inclusion in studies regarding obesity and CVD. Covariate information, including sex, age, race and ethnicity, family educational level (based on the family reference person), family income and the poverty-to-income (PIR) ratio (categorized as ≥ 1.30 or < 1.30), alanine aminotransferase levels, aspartate aminotransferase levels, uric acid levels, self-reported weight one year ago, and history of attempted weight loss and weight loss methods, as well as health history/medical conditions (history of heart disease; history of diabetes; current pregnancy status; and current use of antihypertensive drugs, lipid-lowering drugs, and oral hypoglycemic agents), were obtained through baseline survey questionnaires and laboratory test data. Due to the different definitions of obesity in adults and children, we categorized the participants into four groups based on their BMI [31, 32]: > 18 years old, underweight (< 18.5 kg/m^2^), normal weight (18.5 to < 25 kg/m^2^), overweight (25.0 to < 30) kg/m^2^, and obesity (≥ 30.0 kg/m^2^); and 16–18 years old, underweight (below the 5th percentile for the same age and sex) and normal weight (between the 5th and 85th percentiles for the same age and sex), overweight (between the 85th and 95th percentiles for the same age and sex), and obesity (above or equal to the 95th percentile for the same age and sex). Subsequently, the study participants were classified into five different body shape patterns based on WC: underweight, normal weight, overweight/general obesity (OGO) (male WC < 102 cm, female WC < 88 cm, with an elevated BMI), abdominal obesity (male WC ≥ 102 cm, female WC ≥ 88 cm, with normal BMI), and compound obesity (presence of both general obesity and abdominal obesity) [33].

### CVH scores

The CVH scores include health behaviors such as diet, physical activity, nicotine exposure, and sleep health as well as health factors such as BMI, blood lipids, blood glucose, and blood pressure. In the NHANES, trained healthcare technicians recorded participants’ anthropometric data at mobile examination centers. The data for all the variables were collected across multiple NHANES survey cycles. Dietary scores are determined based on dietary intake data, which are assessed through two 24-h dietary recalls and dietary studies utilizing nutrition databases. Physical activity scores are determined based on self-reported physical activity levels from the Global Physical Activity Questionnaire (GPAQ), while sleep scores are determined based on self-reported average sleep duration during weekdays and nonworking days. Blood lipid scores are determined based on non-HDL levels, which are derived from the difference between total cholesterol levels measured using enzymatic methods and high-density lipoprotein (HDL) cholesterol levels directly measured using immunoturbidimetric assay. Blood glucose scores are determined based on blood glucose or glycated hemoglobin levels, as well as self-reported history of diabetes and oral antidiabetic medication usage. Blood pressure scores are determined based on the level of blood pressure, which is measured multiple times by trained professionals using a mercury sphygmomanometer. Systolic and diastolic pressures are calculated based on up to four readings. If multiple measurements were available, the average of the remaining measurements was used; otherwise, the first measurement was used. The scoring algorithm for each statistical data point is provided in Supplementary Table S1 and presidential advisory [26]. In the preliminary analysis of the present study, records of nicotine exposure related to nicotine delivery systems were excluded from the total score for accuracy because these records were collected only after 2013. The scores of each of the 7 CVH indicators were averaged (unweighted), excluding the nicotine exposure score, to calculate the total score, which ranged from 0 to 100. According to the recommendations of the AHA, the classification of CVH status was as follows: scores between 0 and 49 were classified as low CVH, scores between 50 and 79 were classified as moderate CVH, and scores between 80 and 100 were classified as high CVH. The main outcome variable of this study was the summary of the scores of the 7 CVH indicators, either as a continuous variable or a categorical variable (high, moderate, or low).

### Statistical analyses

Baseline features are presented as the mean and standard deviation or median (interquartile range [IQR]) of continuous variables and as the proportion of categorical variables, with CVH based on overall CVH scores (high, moderate, or low CVH). Differences between features were compared using analyses of variance (ANOVAs) or Chi-square tests, as appropriate. Univariate and multivariate linear regression analyses were employed to assess the correlation between the weight loss percentage and the CVH scores. Covariates were selected based on medical knowledge and impact on the main effect estimate (≥ 10%). The multivariable models were adjusted for sex, age, race, family PIR, educational attainment of the head of household, attempted weight loss in the past year, ALT levels, AST levels, and uric acid levels. Logistic regression models were used to calculate the odds ratios (ORs) and 95% confidence intervals (CIs) for the correlation between the weight loss percentage and CVH category. Restricted cubic splines (RCSs) were successively used to evaluate the nonlinear relationship between the weight loss percentage and the CVH scores as well as to simultaneously assess the nonlinear dose‒response relationship between WC and poor CVH risk. The discriminatory ability of WC in identifying poor CVH risk was evaluated using receiver operating characteristic (ROC) curves. Subgroup analyses were conducted using linear regression models and are presented using forest plots. In the case of categorical variables, the missing values were imputed as the most frequent value among those with data. Missing values were imputed as “college or above” for the categorical variable educational attainment of the household head, for example. The missing values for continuous variables were imputed as the median.

To examine the robustness of the associations, we conducted several sensitivity analyses. We performed analyses with a subset of participants aged ≥ 20 years (n = 11,141) after excluding pregnant individuals (*n* = 90) and those with a history of CVD (congestive heart failure, coronary heart disease, angina pectoris, myocardial infarction, or stroke; *n* = 783) (Supplementary Tables S5, S6). Additionally, we conducted a reevaluation of the participants (*n* = 6259) from the 2013–2018 NHANES cycles. This reevaluation involved recalculating the cardiovascular health (CVH) score by incorporating the factor of nicotine exposure and excluding missing data (*n* = 1818) associated with nicotine exposure (see Additional file 7, Supplementary Tables S7, S8).

The statistical software package R 3.3.2 (http://www.R-project.org, The R Foundation) was used for all analyses. A two-tailed test was used, and a result was considered statistically significant when *p* < 0.05.

## Results

### Demographic characteristics of participants

Regarding the baseline characteristics of the research population, divided into groups according to CVH (as shown in Table 1), among a total of 12,835 participants (mean age: 42.7 ± 19.0 years, 53.1% male), 5.5%, 61.7%, and 32.8% had low, moderate, and high CVH, respectively. Compared to participants with lower CVH scores, individuals with higher CVH scores were more likely to be younger, female, have higher incomes, have higher educational attainment of the head of household, be non-Hispanic White in ethnicity, have a normal body weight, have a history of attempting weight loss, have lower liver function indicators, have lower uric acid levels, have a smaller WC and have a smaller weight loss percentage. Notably, 42.3% of the participants attempted weight loss, among whom 37.2% achieved weight reduction, but only 11.6% experienced weight loss greater than 5%. Within the group of participants who had lost weight, those with a weight loss percentage of 0–5% more often exhibited healthier eating habits and engaged in more physical activity (Supplementary Table S3).

**Table 1:** Baseline characteristics of participants classified by CVH category

Characteristic	Total (*n* = 12,835)	Low CVH (*n* = 709)	Moderate CVH (*n* = 7920)	High CVH (*n* = 4206)	*P* value
Sex^a^					< 0.001
Male	6815 (53.1)	424 (59.8)	4460 (56.3)	1931 (45.9)	
Female	6020 (46.9)	285 (40.2)	3460 (43.7)	2275 (54.1)	
Age^a^, years	42.7 ± 19.0	48.2 ± 17.4	44.5 ± 19.6	38.3 ± 17.3	< 0.001
Race^a^					< 0.001
Mexican American	1778 (13.9)	99 (14)	1176 (14.8)	503 (12)	
Non-Hispanic White	5541 (43.2)	249 (35.1)	3315 (41.9)	1977 (47)	
Non-Hispanic Black	2523 (19.7)	236 (33.3)	1733 (21.9)	554 (13.2)	
Other race	2993 (23.3)	125 (17.6)	1696 (21.4)	1172 (27.9)	
Family poverty–income ratio^a^					< 0.001
< 1.3	3083 (26.1)	216 (32.5)	2006 (27.6)	861 (22.2)	
≥ 1.3	8747 (73.9)	448 (67.5)	5274 (72.4)	3025 (77.8)	
Educational attainment of household head^a^					< 0.001
Below high school	2889 (22.5)	226 (31.9)	1953 (24.7)	710 (16.9)	
High-school graduate	2821 (22.0)	175 (24.7)	1895 (23.9)	751 (17.9)	
College or above	7125 (55.5)	308 (43.4)	4072 (51.4)	2745 (65.3)	
ALT^b^, (U/L)	20.0 (16.0, 27.0)	24.0 (18.0, 35.0)	21.0 (16.0, 29.0)	19.0 (15.0, 24.0)	< 0.001
AST^b^, U/L	23.0 (19.0, 27.0)	24.0 (19.0, 29.0)	23.0 (19.0, 28.0)	23.0 (19.0, 27.0)	< 0.001
Uric acid^a^, mg/dL	5.4 ± 1.4	6.2 ± 1.5	5.5 ± 1.4	4.9 ± 1.2	< 0.001
BMI^a^, kg/m^2^	27.5 ± 6.2	35.1 ± 7.2	28.6 ± 6.1	24.1 ± 3.7	< 0.001
Waist Circumference, cm	94.4 ± 15.8	113.5 ± 16.7	97.5 ± 15.3	85.3 ± 10.4	< 0.001
Obesity patterns^a^					< 0.001
Normal weight	4564 (35.6)	18 (2.5)	1971 (24.9)	2575 (61.2)	
Underweight	226 ( 1.8)	12 (1.7)	133 (1.7)	81 (1.9)	
Overweight /general obesity	4359 (34.0)	127 (17.9)	3041 (38.4)	1191 (28.3)	
Abdominal obesity	313 ( 2.4)	4 (0.6)	154 (1.9)	155 (3.7)	
Compound obesity	3373 (26.3)	548 (77.3)	2621 (33.1)	204 (4.9)	
Attempts to lose weight in past year^a^					< 0.001
Yes	7410 (57.7)	310 (43.7)	4383 (55.3)	2717 (64.6)	
No	5425 (42.3)	399 (56.3)	3537 (44.7)	1489 (35.4)	
Weight from one year prior^b^, kg	73.5 (62.6, 86.2)	94.8 (81.6, 108.4)	77.1 (65.8, 88.5)	65.8 (57.6, 74.8)	< 0.001
Current weight^a^, kg	78.3 ± 19.8	101.0 ± 24.3	81.5 ± 19.6	68.5 ± 13.2	< 0.001
Value of weight loss^b^, kg	− 1.3 (− 5.0, 1.3)	− 2.5 (− 9.7, 1.3)	− 1.6 (− 5.6, 1.3)	− 0.7 (− 3.5, 1.5)	< 0.001
Percentage of weight loss(%)^b^	− 1.8 (− 6.8, 1.8)	− 2.8 (− 10.1, 1.4)	− 2.1 (− 7.5, 1.6)	− 1.0 (− 5.3, 2.2)	< 0.001
Percentage degree of weight loss(%)^a^					< 0.001
< 0	8057 (62.8)	478 (67.4)	5101 (64.4)	2478 (58.9)	
0–5	3290 (25.6)	157 (22.1)	1908 (24.1)	1225 (29.1)	
5.1–10	1016 (7.9)	45 (6.3)	606 (7.7)	365 (8.7)	
10.1–15	322 (2.5)	15 (2.1)	208 (2.6)	99 (2.4)	
15.1–20	82 (0.6)	9 (1.3)	50 (0.6)	23 (0.5)	
> 20	68 (0.5)	5 (0.7)	47 (0.6)	16 (0.4)	
CVH score^a^	68.3 ± 10.9	44.4 ± 4.8	66.7 ± 7.4	84.0 ± 3.6	< 0.001

CVH cardiovascular health (excluding nicotine exposure component); low CVH 0–49; moderate CVH 50–79; high CVH 80–100; ALT alanine aminotransferase; AST glutamic transaminase

^a^Continuous variables are presented as mean ± SD; categorical variables are presented as N(%)

^b^Those characteristics were presented as median (IQR)

### Association between weight loss percentage and CVH scores across individuals with various obesity patterns

Table 2 presents the relationship between the weight loss percentage and the CVH scores across individuals with various obesity patterns. Among all participants, 4564 were normal weight (35.6%), 226 were underweight (1.8%), 4359 had OGO (34.0%), 313 had abdominal obesity (2.4%), and 3373 had compound obesity (26.3%). After adjusting for potential confounding factors, a 1% decrease in body weight was associated with a CVH score increase of 0.18 points. Additionally, within the OGO group, there was an increase of 0.09 points in the CVH score with a 1% decrease in body weight. Compared to those with a weight loss percentage < 0%, participants with weight loss percentages of 0–5% and 5.1–10% demonstrated an improved CVH score, with β values of 2.85 (95% CI 2.32–3.38) and 2.55 (95% CI 1.69–3.4), respectively. Furthermore, within different obesity pattern groups, participants with a weight loss percentage of 0–5% exhibited an increased CVH score in the normal weight and OGO groups compared to those with a weight loss percentage < 0%, with β values of 1.45 (95% CI 0.7–2.19) and 1.22 (95% CI 0.46–1.97), respectively. However, participants with a weight loss percentage of 15.1–20% exhibited decreased CVH scores in the normal weight, abdominal obesity, and compound obesity groups, with β values of − 3.21 (95% CI − 6.44 to 0.03), − 14.15 (95% CI: − 23.72 to − 4.59), and − 6.38 (95% CI − 11.79 to − 0.97), respectively.

**Table 2:** Association between weight loss percentage and CVH scores across individuals with various obesity patterns

Variable	*n*	Univariate model		Multivariate model	
		β (95% CI)	*P* value	β (95% CI)	*P* value
Total					
Per 1% decrease	12,835	0.16 (0.14 to 0.19)	< 0.001	0.18 (0.15 to 0.2)	< 0.001
Percentage degree of weight loss (%)					
< 0	8057	0(Ref)		0 (Ref)	
0–5	3290	2.21 (1.65 to 2.76)	< 0.001	2.85 (2.32 to 3.38)	< 0.001
5.1–10	1016	1.95 (1.06 to 2.84)	< 0.001	2.55 (1.69 to 3.4)	< 0.001
10.1–15	322	0.37 (− 1.15 to 1.88)	0.636	0.48 (− 0.97 to 1.93)	0.513
15.1–20	82	− 2.07 (− 5.03 to 0.89)	0.171	− 1.4 (− 4.19 to 1.38)	0.324
> 20	68	− 2.26 (− 5.51 to 0.99)	0.173	− 1.4 (− 4.53 to 1.73)	0.381
Normal weight					
Per 1% decrease	4564	− 0.04 (− 0.08 to 0)	0.051	0.04 (− 0.01 to 0.08)	0.104
Percentage degree of weight loss (%)					
< 0	2423	0 (Ref)		0 (Ref)	
0–5	1355	0.5 (− 0.23 to 1.23)	0.176	1.45 (0.7 to 2.19)	< 0.001
5.1–10	532	− 2.14 (− 3.17 to − 1.11)	< 0.001	− 0.04 (− 1.09 to 1.01)	0.938
10.1–15	175	− 4.11 (− 5.79 to − 2.43)	< 0.001	− 1.2 (− 2.9 to 0.5)	0.167
15.1–20	43	− 5.41 (− 8.72 to − 2.1)	0.001	− 3.21 (− 6.44 to 0.03)	0.052
> 20	36	− 7.75 (− 11.36 to − 4.14)	< 0.001	− 4.96 (− 8.47 to − 1.45)	0.006
Underweight					
Per 1% decrease	226	− 0.15 (− 0.43 to 0.13)	0.295	0.04 (− 0.26 to 0.33)	0.805
Percentage degree of weight loss (%)					
< 0	78	0 (Ref)		0 (Ref)	
0–5	70	2.07 (− 2.69 to 6.84)	0.395	1.06 (− 3.56 to 5.68)	0.653
5.1–10	39	− 0.1 (− 5.78 to 5.57)	0.972	1.58 (− 3.98 to 7.14)	0.578
10.1–15	28	− 3.18 (− 9.56 to 3.19)	0.329	0.7 (− 5.77 to 7.16)	0.832
15.1–20	6	− 0.26 (− 12.51 to 12)	0.967	1.46 (− 11.16 to 14.09)	0.82
> 20	5	− 16.52 (− 29.87 to − 3.17)	0.016	− 11.11 (− 24.53 to 2.31)	0.106
Overweight/general obesity					
Per 1% decrease	4359	0 (− 0.04 to 0.04)	0.983	0.09 (0.05 to 0.13)	< 0.001
Percentage degree of weight loss (%)					
< 0	2792	0(Ref)		0 (Ref)	
0–5	1173	− 0.03 (− 0.79 to 0.74)	0.947	1.22 (0.46 to 1.97)	0.002
5.1–10	296	− 1.84 (− 3.19 to − 0.5)	0.007	− 0.21 (− 1.52 to 1.1)	0.753
10.1–15	65	− 1.28 (− 4.03 to 1.48)	0.363	0.17 (− 2.49 to 2.82)	0.903
15.1–20	16	− 2.4 (− 7.9 to 3.11)	0.393	− 0.6 (− 5.9 to 4.69)	0.824
> 20	17	3.27 (− 2.07 to 8.61)	0.23	5.36 (0.23 to 10.5)	0.041
Abdominal obesity					
Per 1% decrease	313	− 0.22 (− 0.43 to − 0.02)	0.032	− 0.05 (− 0.24 to 0.15)	0.655
Percentage degree of weight loss (%)					
< 0	211	0 (Ref)		0 (Ref)	
0–5	71	− 3.6 (− 6.65 to − 0.55)	0.021	− 1.4 (− 4.33 to 1.53)	0.351
5.1–10	0				
10.1–15	26	− 9.01 (− 13.64 to − 4.39)	< 0.001	− 4.31 (− 8.83 to 0.21)	0.062
15.1 to 20	5	− 19.4 (− 29.47 to − 9.34)	< 0.001	− 14.15 (− 23.72 to − 4.59)	0.004
> 20	0				
Compound obesity					
Per 1% decrease	3373	− 0.03 (− 0.06 to 0)	0.053	0 (− 0.03 to 0.03)	0.843
Percentage degree of weight loss (%)					
< 0	2553	0 (Ref)		0 (Ref)	
0–5	621	− 1.27 (− 2.31 to − 0.24)	0.016	− 0.11 (− 1.12 to 0.91)	0.836
5.1–10	123	− 1.38 (− 3.52 to 0.75)	0.204	− 0.43 (− 2.48 to 1.62)	0.682
10.1–15	49	− 2.77 (− 6.1 to 0.57)	0.104	− 2.49 (− 5.69 to 0.72)	0.129
15.1–20	17	− 8.3 (− 13.92 to − 2.67)	0.004	− 6.38 (− 11.79 to − 0.97)	0.021
> 20	10	− 9.59 (− 16.92 to − 2.27)	0.01	− 9.79 (− 16.82 to − 2.77)	0.006

CVH cardiovascular health (excluding nicotine exposure component); multivariate model was adjusted for age, sex, race, family PIR, the educational attainment of household head, attempts to lose weight in past year, ALT, AST, uric acid

**Fig. 2:**
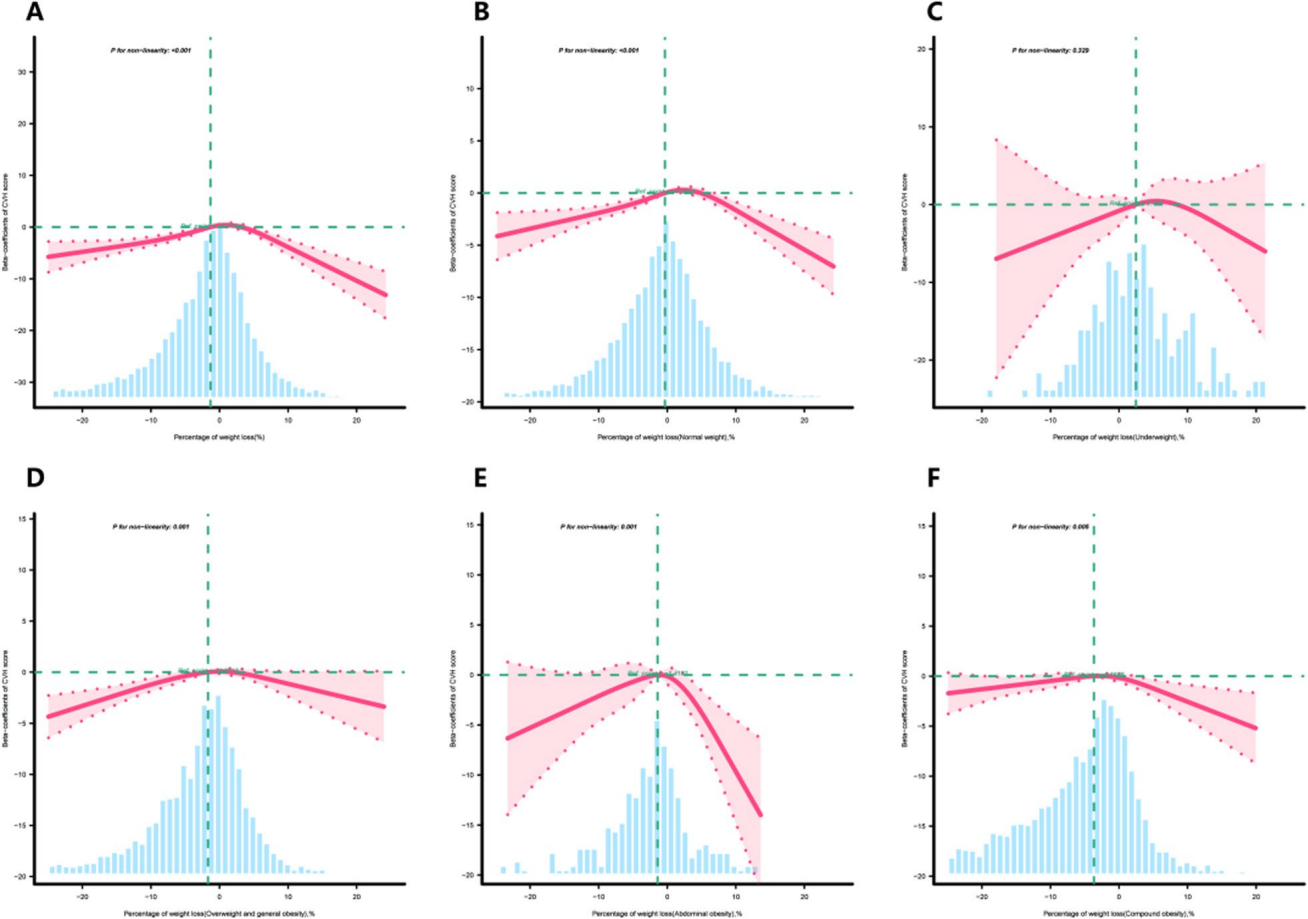
Association between weight loss percentage and CVH scores across individuals with various obesity patterns beta-coefficients. **A** All participants, **B** normal weight, **C** underweight, **D** overweight and general obesity, **E** abdominal obesity, **F** compound obesity. Solid and dashed lines represent the predicted value and 95% confidence intervals. The models were adjusted for sex, age, race, family poverty–income ratio, educational attainment of household head, attempts to lose weight in past year, ALT, AST, and uric acid

Multivariable adjusted RCS analysis revealed a significant inverted U-shaped association between the weight loss percentage and the CVH scores, as depicted in Fig. 2. Specifically, the regression coefficient related to the CVH scores was highest when the weight loss percentage was 3% (Table 3). Furthermore, within different obesity pattern groups, we observed similar relationships between the normal weight and OGO groups. The relationship between the weight loss percentage and CVH category is displayed in Supplementary Table S2. For every 1% decrease in weight loss percentage, the odds of moderate CVH increased by 3%, and the risk of poor CVH increased by 4%. Compared to individuals with a weight loss percentage < 0%, individuals with weight loss percentages of 0–5% and 5.1–10% had adjusted ORs for high CVH of 1.87 (95% CI 1.5–2.32) and 1.83 (95% CI 1.28–2.61), respectively.

**Table 3:** Threshold effect analysis of the relationship between weight loss percentage and CVH scores

Percentage of weight loss (%)	Adjusted model	
	β (95% CI)	*P* value
< 3.0	0.313 (0.27–0.356)	< 0.001
≥ 3.0	− 0.214(− 0.469–0.041)	0.1
Logarithmic likelihood ratio test *P* value		< 0.001

CVH cardiovascular health (excluding nicotine exposure component); adjusted model was adjusted for age, sex, race, family PIR, the educational attainment of household head, attempts to lose weight in past year, ALT, AST, and uric acid. Only 95% of the data are displayed

### Association between the weight loss percentage and aspects of CVH

**Fig. 3:**
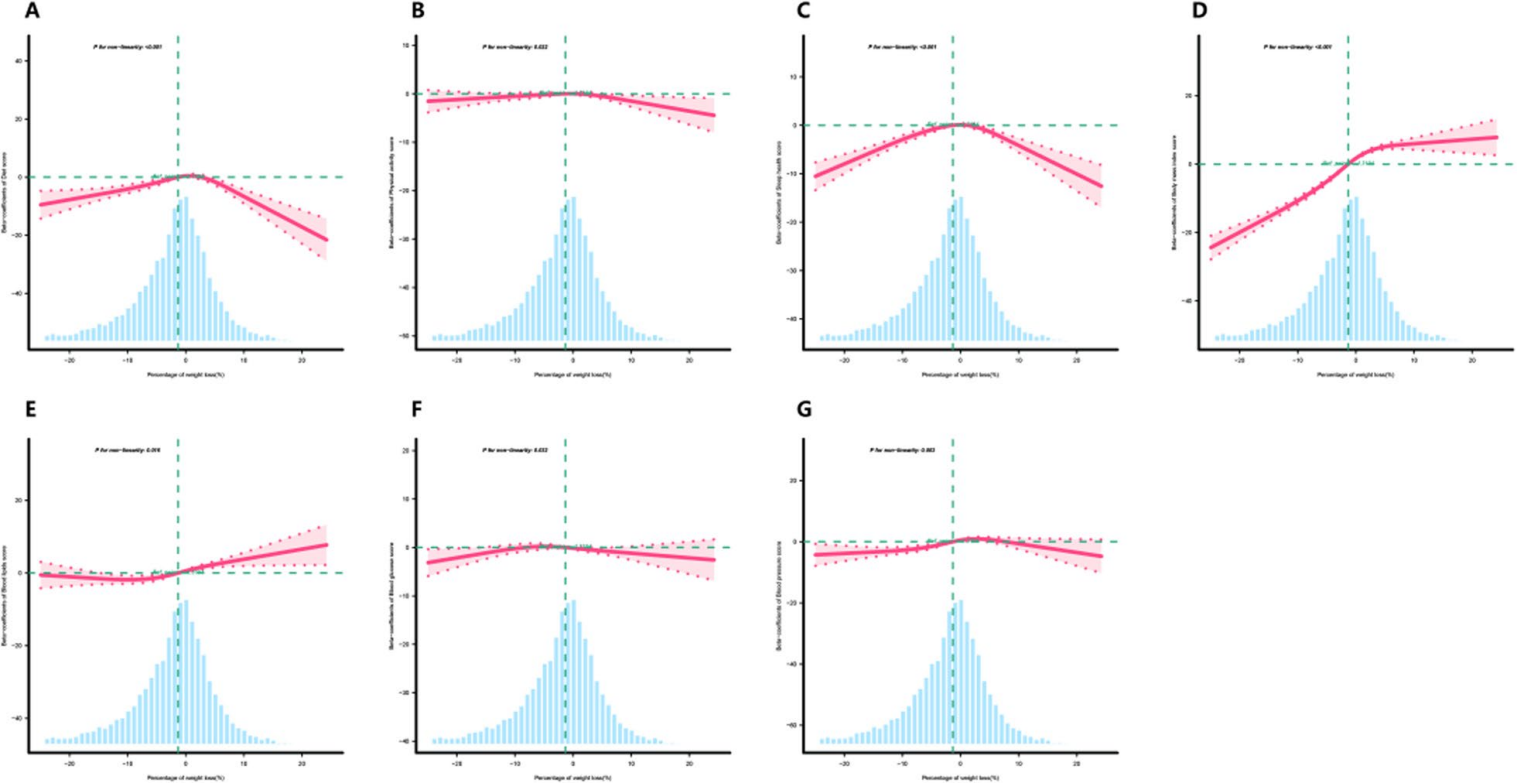
Association between the weight loss percentage and aspects of CVH beta-coefficients. **A** Diet, **B** physical activity, **C** sleep health, **D** body mass index, **E** blood lipids, **F** blood glucose, **G** blood pressure. Solid and dashed lines represent the predicted value and 95% confidence intervals. The models were adjusted for sex, age, race, family poverty–income ratio, educational attainment of household head, attempts to lose weight in past year, ALT, AST, and uric acid

After establishing the relationship between the weight loss percentage and overall CVH scores, we next examined specific aspects of CVH. According to our findings (Supplementary Table S4 and Fig. 3), there was a positive correlation between weight loss percentage and the scores for sleep, BMI, blood lipid levels, and blood pressure. Specifically, for every 1% decrease in weight, the corresponding scores increased by 0.09, 0.66, 0.18, and 0.08 points, respectively. Furthermore, the results indicated that participants who achieved a weight loss percentage of 0–5% demonstrated improvements in various cardiovascular scores (except for blood glucose) compared to those with a weight loss percentage < 0%. At a weight loss percentage of 5.1–10%, the scores for blood lipid levels and blood pressure showed further increases. However, when the weight loss percentage reached 10–15%, there was a decrease in scores, including those for diet, sleep health, and blood glucose. Overall, moderate weight loss appeared to lead to better CVH outcomes than severe weight loss.

### Stratified analyses by potential effect modifiers

**Fig. 4:**
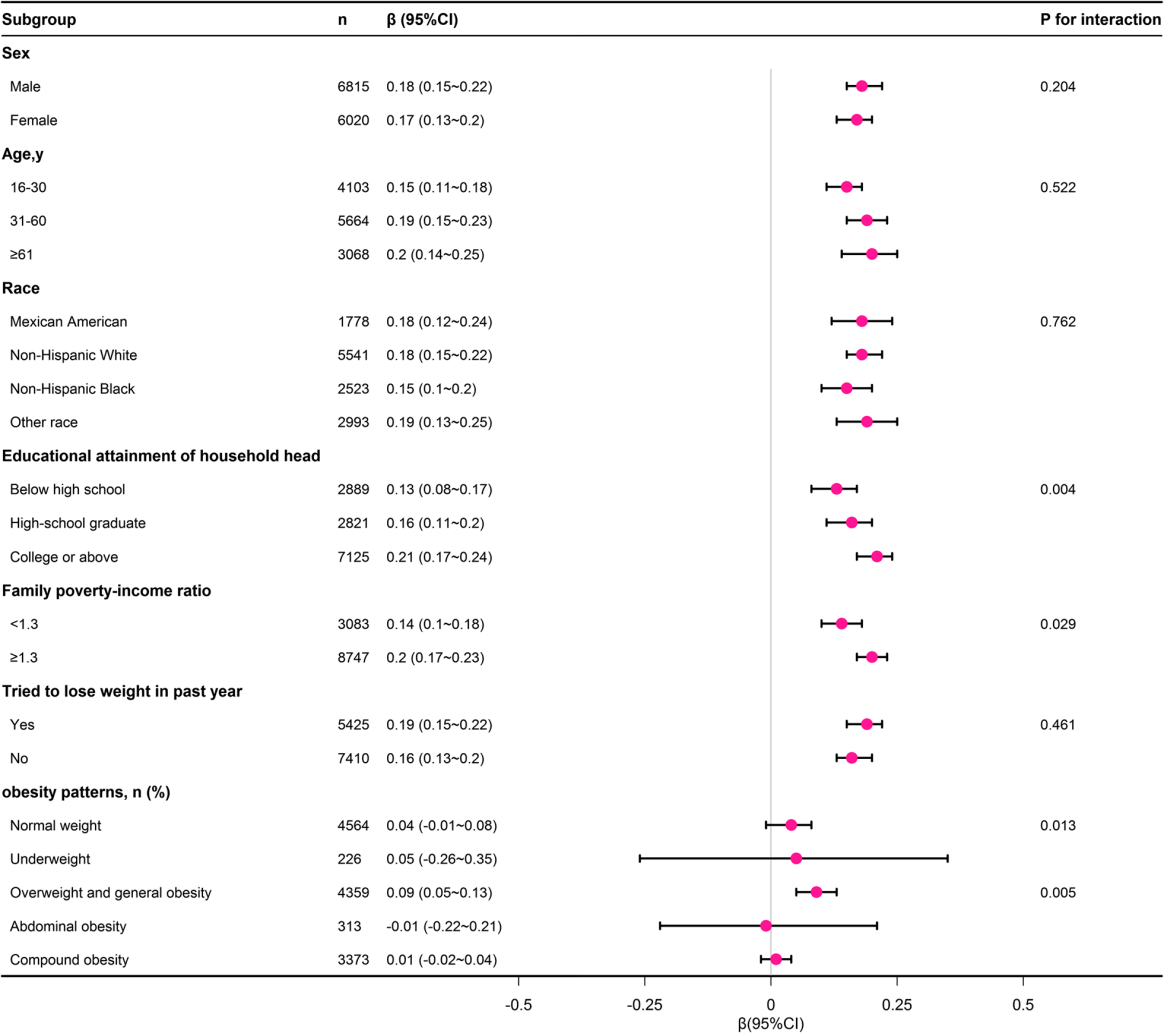
Subgroup analysis of the association between weight loss percentage and CVH scores. Each stratification was adjusted for sex, age, race, family poverty–income ratio, educational attainment of household head, tried to lose weight in past year, ALT, AST, and Uric acid except the stratification factor itself. Circles indicate β, with horizontal lines indicating 95% CIs

Figure 4 presents the results of subgroup analyses, revealing a consistent correlation between the weight loss percentage and the CVH scores across various subgroups, including age, sex, race, and attempts to lose weight in the past year. Notably, this correlation was found to be more pronounced within subgroups defined by economic income, educational level of the head of household, and OGO.

### Association between WC and the risk of poor CVH

**Fig. 5:**
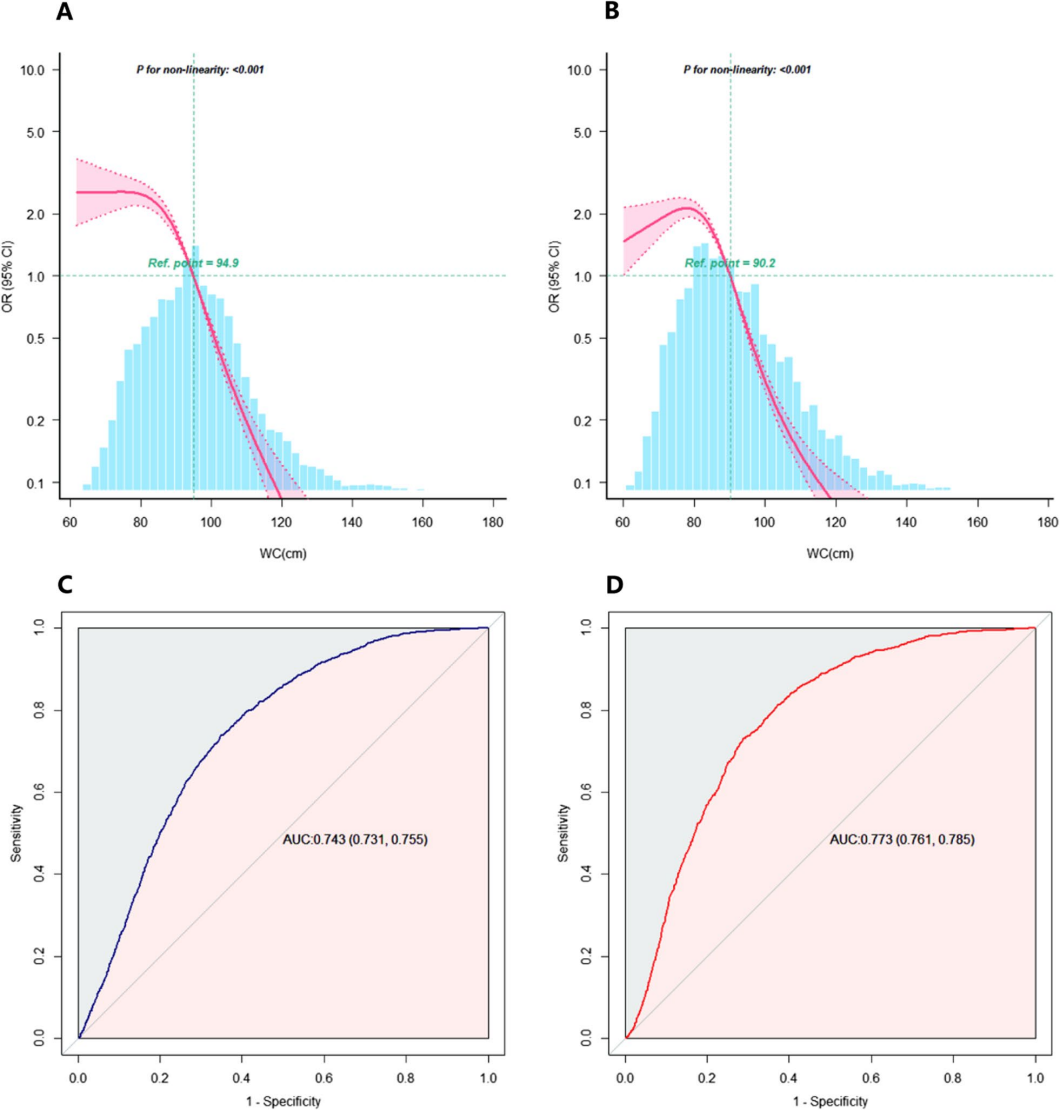
RCS analysis between WC and the risk of poor CVH and the ROC curve. **A** RCS analysis for the association between male WC and the risk of poor CVH. **B** RCS analysis for the association between female WC and the risk of poor CVH. **C** ROC curve of mela WC for poor CVH risk. **D** ROC curve of female WC for poor CVH risk

After full adjustment for variables such as age, race, family PIR, educational attainment of the head of household, history of weight loss attempts, ALT levels, AST levels, and uric acid levels, RCS analysis revealed a nonlinear relationship between WC and the risk of poor CVH. Beyond specific thresholds (94.9 cm for males and 90.2 cm for females), the risk of poor CVH increased rapidly. ROC analysis indicated that WC exhibited good discriminatory ability regarding the risk of poor CVH, with area under the curve (AUC) values of 0.743 (95% CI 0.731–0.755) for males and 0.773 (95% CI 0.761–0.785) for females (Fig. 5).

### Sensitivity analyses

To examine the robustness of the association, we conducted several sensitivity analyses. First, we focused exclusively on analyzing the CVH scores of participants aged ≥ 20 years while excluding individuals with a history of baseline cardiac disease or pregnancy. Notably, we observed a persistently significant correlation between the weight loss percentage and the CVH scores (Supplementary Tables S5, S6). Finally, we conducted a reevaluation of the Cardiovascular Health (CVH) scores for the 2013–2018 NHANES cycles. In this analysis, we incorporated nicotine exposure as an additional scoring factor and performed a subsequent analysis in conjunction with the weight loss percentage. The obtained results reaffirmed our previous conclusions, demonstrating consistency (Supplementary Tables S5, S8).

## Discussion

In this cross-sectional study, we conducted a preliminary investigation into the relationship between the percentage of weight reduction and CVH scores using NHANES data. The results revealed an inverted U-shaped relationship between the weight loss percentage and the CVH scores, with moderate weight reduction (0–10%, optimal value at 3%) potentially improving CVH scores, particularly in OGO patients. However, excessive weight loss (> 15–20%) did not confer any benefits. This association was robustly supported in the sensitivity analysis. Subgroup analysis further revealed a notably significant correlation between the weight loss percentage and the CVH scores among individuals with OGO. Additionally, high WC tended to be associated with low CVH in individuals with abdominal obesity or compound obesity.

Our findings suggest that there is a positive correlation between the weight loss percentage and the CVH scores, which is consistent with previous studies [34]. Additionally, we discovered a complex relationship between the weight loss percentage and various CVH indicators. First, compared to participants with a weight loss percentage < 0%, those with a weight loss percentage > 0% demonstrated better performance in terms of blood pressure, blood lipid levels, sleep, physical activity, and diet. Multiple studies have indicated that a balanced diet and regular exercise are closely related to weight loss [35–40], with exercise reducing blood pressure and blood lipid levels as well as improving sleep duration and quality [30, 41–47]. Moreover, previous research has shown that weight loss can alleviate insulin resistance and aid in blood sugar control [48–50]. The results from a multicenter trial on diabetes prevention programs revealed that weight loss > 5% can reduce risk factors for CVD, such as abnormal blood lipid levels, hypertension, and diabetes[30]. Furthermore, the likelihood of progressing from impaired glucose tolerance to diabetes was reduced by 58% among patients who achieved a weight loss of 7% [48]. We believe that the discrepancy in research results is due to the widespread use of hypoglycemic medications among diabetic participants in our study, which reduced the impact of weight loss on blood sugar levels. Additionally, we found this positive correlation only within individuals with a weight loss percentage of 0–10%. Once the weight loss percentage exceeded 10–15%, indicators such as exercise, blood pressure, and overall CVH scores became independent of the weight loss percentage, suggesting that individuals had attained a threshold for improvement in blood pressure and CVH. On the other hand, indicators such as diet, sleep, and blood sugar scores exhibit a negative correlation with the weight loss percentage, indicating that substantial weight loss may affect diet and sleep patterns. The results from Supplementary Table S9 can explain this phenomenon, as participants with a weight loss percentage of 0–5% demonstrated better adherence to a healthy diet, exercise, and sleep, with longer sleep durations and better average systolic blood pressure levels. However, participants with a weight loss percentage of 10.1–20% seemed to adopt some less recommended weight loss strategies, such as skipping meals and using weight loss medications, while also exhibiting shorter sleep durations. Prolonged energy restriction and excessive dieting can lead to a decrease in the metabolic rate and loss of muscle mass, which can adversely affect CVH. Excessive weight loss may also result in arrhythmias, hypotension, and electrolyte imbalances, further increasing the risk of CVD. Similar results were observed among different obesity pattern groups, with participants who lost 15.1–20% of their body weight exhibiting a decline in CVH scores in the normal weight, abdominal obesity, and compound obesity groups. The results of a randomized controlled trial showed that excessive weight fluctuations are detrimental to CVH [51]. However, the results of a multicenter trial conducted among patients with type 2 diabetes and BMI > 25 kg/m^2^ showed that weight loss ≥ 10% within the first year was associated with a reduced risk of fatal and nonfatal cardiovascular events after 10 years (HR: 0.79, 95% CI 0.64–0.98) in all participants [52]. Therefore, further research is required to elucidate the potential benefits and drawbacks of substantial weight loss on CVH. Overall, there appeared to be an inverted U-shaped association between the weight loss percentage and the CVH scores (Fig. 2), with the strongest positive effects on CVH observed at a weight loss percentage of 3%. Therefore, moderate weight loss (0–10%, optimal value of 3%) may potentially improve cardiovascular health (CVH) scores, while excessive weight loss (> 15–20%) may not provide any benefits.

Another interesting finding was that among different obesity pattern groups, only participants with normal weight or OGO demonstrated improvements in the CVH scores with a weight loss percentage of 0–5% compared to those who did not exhibit weight loss. In contrast, individuals with other obesity patterns, such as underweight, abdominal obesity, and compound obesity, did not exhibit similar improvements. Furthermore, stratified analysis highlighted a significant association primarily in the OGO group, which may be attributed to population heterogeneity. Specifically, individuals with OGO generally have a higher BMI, and weight loss significantly improves CVH in this population. Similar benefits were observed in individuals with normal weight, such as those with metabolically obese normal weight [53]. Underweight may signal debility, including malnutrition and comorbidities other than cardiovascular complications. However, debility itself increases the risk of CVD and death [54, 55]. Studies have shown an association between underweight and an increased risk of cardiovascular events and death [56–59], so further weight loss does not provide CVH benefits in this group. Additionally, WC is one of the most commonly used anthropometric measures for diagnosing abdominal obesity. Recent research has demonstrated an association between increased WC and increased risk of coronary heart disease, independent of other potential risk factors such as BMI [60]. A large WC indicates an increased risk of cardiac metabolism and is a risk factor for myocardial infarction [44, 61]. Similar manifestations can be observed even in individuals who are not overweight overall (i.e., abdominal obesity without general overweight) [62]. Patients with abdominal obesity have increased risks of CVD, diabetes, hypertension, dyslipidemia, and nonalcoholic fatty liver disease, as well as higher overall mortality rates [63–65]. Furthermore, we investigated the dose‒response relationship between WC and the risk of poor CVH, and the results showed a significant increase in the risk of poor CVH with a larger WC. Thus, for individuals with abdominal obesity, controlling WC is equally important as reducing BMI, and it may even be more beneficial in terms of improving CVH. Additionally, stratified analysis revealed differences in the impact of weight loss on CVH according to income and education level, which may be attributed to the fact that individuals with higher income levels and education levels tend to adopt healthier and more reasonable weight loss strategies [66–68], such as consulting health experts or physicians and choosing healthy and nutritious food options.

## Strength and limits

We utilized data from the NHANES in our study, as the NHANES recruited a representative sample according to age, sex, and race in the United States, thereby enhancing the reliability and generalizability of our findings. However, our study also has certain limitations. First, due to the cross-sectional design, we could not establish causal relationships; thus, further prospective studies are needed to confirm our results. Second, despite considering numerous covariates in our regression models, residual confounding effects from unmeasured or unknown factors could not be completely eliminated. Furthermore, utilizing self-reported weight data from the previous year to ascertain weight loss may introduce the possibility of misreporting. However, previous research reports have shown that weight history recall is relatively stable and exhibits minimal deviation [69], thereby contributing to a certain degree of reliability in our research findings. Last, we solely relied on a nationally representative sample from the United States, but there are significant differences among racial groups regarding dietary habits, activity levels, sleep patterns, genetic variations, etc., which limit the generalizability of our findings to populations in other countries. In conclusion, given these limitations, it is imperative to validate our findings in subsequent studies.

## Conclusions

Based on our research findings, it is evident that moderate weight loss may have a greater impact on improving CVH than excessive weight loss. These results emphasize the importance of targeted weight loss interventions for optimal health outcomes. Furthermore, our study indicates that in individuals with abdominal obesity or compound obesity, focusing on reducing WC may be more meaningful than solely reducing BMI. These novel insights not only contribute to the existing body of knowledge, but also underscore the necessity of tailored strategies to address specific obesity phenotypes. Further investigation is warranted to explore the broader applicability of our findings and delve into unanswered questions, ultimately deepening our understanding of the complex relationship between weight loss, cardiovascular health, and different obesity patterns.

## What is already known on this subject?

Regarding the relationship between weight loss and cardiovascular health, the existing body of research has provided limited evidence, with the majority of studies focusing on populations with preexisting conditions such as hypertension and diabetes. However, there remains a paucity of exploration specifically targeting individuals with distinctive body types.

## What this study adds?

Moderate weight loss may have a more significant impact on improving cardiovascular health compared to excessive weight loss. Additionally, in individuals with abdominal obesity or compound obesity, reducing waist circumference carries greater importance in enhancing CVH. This study has expanded our understanding of the relationship between different obesity patterns and cardiovascular health while emphasizing the necessity of developing personalized strategies for specific obesity phenotypes. These findings provide a feasible basis for future intervention measures.

### Supplementary Information

Below is the link to the electronic supplementary material.

Supplementary file1 (DOCX 20 KB)

Supplementary file2 (DOCX 14 KB)

Supplementary file3 (DOCX 23 KB)

Supplementary file4 (DOCX 15 KB)

Supplementary file5 (DOCX 23 KB)

Supplementary file6 (DOCX 14 KB)

Supplementary file7 (DOCX 23 KB)

Supplementary file8 (DOCX 14 KB)

Supplementary file9 (DOCX 19 KB)

Supplementary file10 (DOCX 52 KB)

## Data Availability

The datasets generated during and analyzed during the current study are available from the corresponding author on reasonable request.
