# Supplementary material for: The inverted U-shaped relationship between weight loss percentage and cardiovascular health scores
Source: Eat Weight Disord. 2023 Oct 24;28(1):87. doi: 10.1007/s40519-023-01619-3 (PMC10598164; doi:10.1007/s40519-023-01619-3)
Supplement: Supplementary file 3 — Supplementary file3 (DOCX 23 KB) [file 40519_2023_1619_MOESM3_ESM.docx]

**Table 4** Association between weight loss percentage and CVH category

| Variable | Unadjusted Model | | Adjusted Model | |
| --- | --- | --- | --- | --- |
|  | Moderate versus low CVH | High versus low CVH | Moderate versus low CVH | High versus low CVH |
| Percentage of weight loss(%) | 1.01 (1.01~1.02)* | 1.04 (1.03~ 1.04)* | 1.01 (1~1.02)* | 1.04 (1.03~ 1.05)* |
| Percentage degree of weight loss(%) |  | | | |
| <0 | 1 (Ref) | 1 (Ref) | 1 (Ref) | 1 (Ref) |
| 0~5 | 1.14 (0.94~1.37) | 1.5 (1.24~ 1.83)* | 1.22 (1~1.5) | 1.87 (1.5~ 2.32)* |
| 5.1~10 | 1.26 (0.92~1.73) | 1.56 (1.13~ 2.16)* | 1.23 (0.88~1.73) | 1.83 (1.28~ 2.61)* |
| 10.1~15 | 1.3 (0.76~2.21) | 1.27 (0.73~ 2.21) | 1.24 (0.7~2.19) | 1.29 (0.7~ 2.37) |
| 15.1~20 | 0.52 (0.25~1.07) | 0.49 (0.23~ 1.07) | 0.5 (0.24~1.06) | 0.49 (0.21~ 1.16) |
| >20 | 0.88 (0.35~2.23) | 0.62 (0.23~ 1.69) | 0.85 (0.29~2.45) | 0.74 (0.23~ 2.37) |

~~^[[1]](#footnote-0)^~~

1. CVH cardiovascular health (excluding nicotine exposure component); Adjusted Model was adjusted for age, sex, race, family PIR, the educational attainment of household head, attempts to lose weight in past year, ALT, AST and Uric acid.

   * *P*<0.05. [↑](#footnote-ref-0)
