# Supplementary material for: The inverted U-shaped relationship between weight loss percentage and cardiovascular health scores
Source: Eat Weight Disord. 2023 Oct 24;28(1):87. doi: 10.1007/s40519-023-01619-3 (PMC10598164; doi:10.1007/s40519-023-01619-3)
Supplement: Supplementary file 4 — Supplementary file4 (DOCX 15 KB) [file 40519_2023_1619_MOESM4_ESM.docx]

**Supplementary Table 3.** Baseline characteristics of participants classified by percentage degree of weight loss

| Characteristic | Total (n = 12835) | <0% (n = 8057) | 0-5% (n = 3290) | 5.1-10%(n = 1016) | 10.1-15% (n = 322) | 15.1-20% (n = 82) | >20% (n = 68) |
| --- | --- | --- | --- | --- | --- | --- | --- |
| Sex^a^ |  |  |  |  |  |  |  |
| Male | 6815 (53.1) | 3898 (48.4) | 1973 (60) | 639 (62.9) | 208 (64.6) | 54 (65.9) | 43 (63.2) |
| Female | 6020 (46.9) | 4159 (51.6) | 1317 (40) | 377 (37.1) | 114 (35.4) | 28 (34.1) | 25 (36.8) |
| Age^a^,years | 42.7 ± 19.0 | 40.4 ± 18.4 | 47.4 ± 18.9 | 45.5 ± 19.9 | 44.3 ± 20.9 | 43.0 ± 20.2 | 45.6 ± 20.5 |
| Race^a^ |  |  |  |  |  |  |  |
| Mexican American | 1778 (13.9) | 1143 (14.2) | 405 (12.3) | 157 (15.5) | 49 (15.2) | 13 (15.9) | 11 (16.2) |
| Non-Hispanic White | 5541 (43.2) | 3548 (44) | 1432 (43.5) | 388 (38.2) | 117 (36.3) | 24 (29.3) | 32 (47.1) |
| Non-Hispanic Black | 2523 (19.7) | 1547 (19.2) | 601 (18.3) | 222 (21.9) | 107 (33.2) | 30 (36.6) | 16 (23.5) |
| Other race | 2993 (23.3) | 1819 (22.6) | 852 (25.9) | 249 (24.5) | 49 (15.2) | 15 (18.3) | 9 (13.2) |
| Family poverty-income ratio^a^ |  |  |  |  |  |  |  |
| <1.3 | 3083 (26.1) | 1894 (25.4) | 690 (22.8) | 316 (34.6) | 114 (39) | 37 (48.7) | 32 (53.3) |
| ≥1.3 | 8747 (73.9) | 5566 (74.6) | 2340 (77.2) | 596 (65.4) | 178 (61) | 39 (51.3) | 28 (46.7) |
| Educational attainment of household head^a^ |  |  |  |  |  |  |  |
| Below high school | 2889 (22.5) | 1680 (20.9) | 737 (22.4) | 309 (30.4) | 102 (31.7) | 31 (37.8) | 30 (44.1) |
| High-school graduate | 2821 (22.0) | 1727 (21.4) | 739 (22.5) | 240 (23.6) | 84 (26.1) | 17 (20.7) | 14 (20.6) |
| College or above | 7125 (55.5) | 4650 (57.7) | 1814 (55.1) | 467 (46) | 136 (42.2) | 34 (41.5) | 24 (35.3) |
| ALT^a^, (U/L) | 20.0 (16.0, 27.0) | 20.0 (16.0, 28.0) | 20.0 (16.0, 27.0) | 19.0 (15.0, 25.0) | 19.0 (14.0, 24.0) | 17.0 (15.0, 21.0) | 17.0 (15.0, 24.2) |
| AST^a^, U/L | 23.0 (19.0, 27.0) | 23.0 (19.0, 28.0) | 23.0 (20.0, 28.0) | 22.0 (19.0, 26.0) | 22.0 (19.0, 26.8) | 21.0 (19.0, 26.8) | 23.0 (18.0, 27.2) |
| Uric acid^a^, mg/dL | 5.4 ± 1.4 | 5.4 ± 1.4 | 5.4 ± 1.4 | 5.3 ± 1.3 | 5.2 ± 1.3 | 5.3 ± 1.5 | 5.1 ± 1.2 |
| Waist Circumference^a^, cm | 94.4 ± 15.8 | 96.2 ± 16.2 | 92.5 ± 14.4 | 89.1 ± 13.6 | 87.1 ± 15.3 | 91.0 ± 18.5 | 91.3 ± 18.3 |
| Obesity patterns^a^ |  |  |  |  |  |  |  |
| Normal weight | 4564 (35.6) | 2423 (30.1) | 1355 (41.2) | 532 (52.4) | 175 (54.3) | 43 (52.4) | 36 (52.9) |
| Underweight | 226 ( 1.8) | 78 (1) | 70 (2.1) | 39 (3.8) | 28 (8.7) | 6 (7.3) | 5 (7.4) |
| Overweight / general obesity | 4359 (34.0) | 2792 (34.7) | 1173 (35.7) | 296 (29.1) | 65 (20.2) | 16 (19.5) | 17 (25) |
| Abdominal obesity | 313 ( 2.4) | 211 (2.6) | 71 (2.2) | 26 (2.6) | 5 (1.6) | 0 (0) | 0 (0) |
| Compound ­obesity | 3373 (26.3) | 2553 (31.7) | 621 (18.9) | 123 (12.1) | 49 (15.2) | 17 (20.7) | 10 (14.7) |
| Attempts to lose weight in past year^a^ |  |  |  |  |  |  |  |
| Yes | 7410 (57.7) | 4213 (52.3) | 2092 (63.6) | 712 (70.1) | 271 (84.2) | 67 (81.7) | 55 (80.9) |
| No | 5425 (42.3) | 3844 (47.7) | 1198 (36.4) | 304 (29.9) | 51 (15.8) | 15 (18.3) | 13 (19.1) |
| Weight from one year prior^a^, kg | 73.5 (62.6, 86.2) | 72.6 (61.2, 84.8) | 74.8 (63.5, 87.1) | 74.8 (64.7, 84.4) | 74.8 (65.8, 88.5) | 83.0 (72.6, 103.2) | 95.3 (79.7, 108.9) |
| Current weight^a^, kg | 78.3 ± 19.8 | 80.7 ± 20.5 | 75.6 ± 18.1 | 71.2 ± 16.3 | 69.7 ± 17.6 | 74.4 ± 23.5 | 73.0 ± 23.1 |
| Value of weight loss^a^, kg | -1.3 (-5.0, 1.3) | -3.8 (-7.5, -1.6) | 1.4 (0.6, 2.3) | 5.0 (4.1, 6.3) | 9.1 (7.7, 11.2) | 14.4 (12.0, 17.1) | 23.0 (18.2, 29.7) |
| Percentage of weight loss^b^ | -1.8 (-6.8, 1.8) | -5.2 (-10.2, -2.2) | 1.9 (0.9, 3.1) | 6.7 (5.8, 8.0) | 12.0 (10.9, 13.5) | 17.2 (15.8, 18.2) | 24.1 (21.3, 30.1) |
| Life’s Essential factors |  |  |  |  |  |  |  |
| Ideal diet (%)^a^ | 3842 (29.9) | 2336 (29) | 1144 (34.8) | 277 (27.3) | 59 (18.3) | 15 (18.3) | 11 (16.2) |
| Ideal physical activity (%)^a^ | 8382 (65.3) | 5201 (64.6) | 2230 (67.8) | 651 (64.1) | 203 (63) | 54 (65.9) | 43 (63.2) |
| Sleep health^a^, h/day | 7.2 ± 1.4 | 7.2 ± 1.4 | 7.2 ± 1.3 | 7.2 ± 1.5 | 7.0 ± 1.7 | 6.9 ± 1.9 | 6.9 ± 1.8 |
| Body mass index^a^, kg/m^2^ | 27.5 ± 6.2 | 28.4 ± 6.4 | 26.3 ± 5.3 | 24.9 ± 4.9 | 24.4 ± 5.8 | 25.9 ± 7.9 | 26.1 ± 8.6 |
| Non–HDL cholestero^a^l, mg/dl | 134.6 ± 41.8 | 135.7 ± 41.8 | 134.1 ± 40.8 | 133.2 ± 44.5 | 120.5 ± 39.3 | 126.1 ± 40.8 | 127.6 ± 43.4 |
| HbA1c^a^, % | 5.6 ± 0.8 | 5.5 ± 0.7 | 5.6 ± 0.9 | 5.6 ± 1.1 | 5.6 ± 1.1 | 5.9 ± 1.7 | 5.9 ± 1.7 |
| Systolic blood pressure^a^, mmHg | 120.3 ± 16.7 | 119.7 ± 16.2 | 121.3 ± 17.1 | 121.4 ± 17.8 | 121.9 ± 19.2 | 120.1 ± 16.2 | 123.9 ± 18.3 |

^[[1]](#footnote-0)^

1. ALT alanine aminotransferase; AST glutamic transaminase.

   ^a^ Continuous variables are presented as mean (SD); categorical variables are presented as N (%).

   ^b^ Those characteristics were presented as median (IQR). [↑](#footnote-ref-0)
