# Supplementary material for: The inverted U-shaped relationship between weight loss percentage and cardiovascular health scores
Source: Eat Weight Disord. 2023 Oct 24;28(1):87. doi: 10.1007/s40519-023-01619-3 (PMC10598164; doi:10.1007/s40519-023-01619-3)
Supplement: Supplementary file 5 — Supplementary file5 (DOCX 23 KB) [file 40519_2023_1619_MOESM5_ESM.docx]

**Supplementary Table 4.** Association between the weight loss percentage and aspects of CVH

| Variable | **Behavioral CVH** | | | **Biological CVH** | | | |
| --- | --- | --- | --- | --- | --- | --- | --- |
|  | Diet | Physical activity | Sleep health | Body mass index | Blood lipids | Blood glucose | Blood pressure |
| Per 1 % decrease | 0.05 (-0.02~0.13) | 0.03 (-0.01~0.07) | 0.09 (0.04~0.14)* | 0.66 (0.61~0.71)* | 0.18 (0.13~0.24)* | 0.03 (-0.01~0.07) | 0.08 (0.03~0.13)* |
| Percentage degree of weight loss(%) |  |  |  |  |  |  |  |
| <0 | 0(Ref) | 0(Ref) | 0(Ref) | 0(Ref) | 0(Ref) | 0(Ref) | 0(Ref) |
| 0-5 | 2.44 (0.86~4.01)* | 0.97 (0.05~1.88)* | 1.63 (0.54~2.72)* | 8.2 (7.02~9.37)* | 3.49 (2.26~4.72)* | 0.07 (-0.84~0.98) | 2.15 (0.99~3.3)* |
| 5.1-10 | -1.78 (-4.31~0.74) | 0.33 (-1.14~1.8) | 0.79 (-0.96~2.54) | 13.37 (11.49~15.25)* | 2.3 (0.33~4.28)* | 0.89 (-0.58~2.35) | 2.09 (0.23~3.94)* |
| 10.1-15 | -8.42 (-12.71~-4.13)* | -0.91 (-3.41~1.58) | -3.51 (-6.47~-0.54)* | 7.67 (4.48~10.87)* | 8.93 (5.58~12.28)* | 0.04 (-2.45~2.52) | 1.21 (-1.94~4.35) |
| 15.1-20 | -4.29 (-12.69~4.12) | 0.36 (-4.53~5.25) | -5.58 (-11.39~0.24)* | 6.8 (0.54~13.07)* | 3.53 (-3.04~10.1) | -8.27 (-13.14~-3.41)* | 3.12 (-3.04~9.29) |
| >20 | -6.79 (-15.92~2.35) | -4.5 (-9.81~0.82) | -6.06 (-12.38~0.25)* | 6.37 (-0.43~13.18) | 6.11 (-1.03~13.25) | -1.05 (-6.34~4.23) | -2.8 (-9.49~3.9) |

^[[1]](#footnote-0)^

1. CVH cardiovascular health (excluding nicotine exposure component); The Model was adjusted for age, sex, race, family PIR, the educational attainment of household head, attempts to lose weight in past year, ALT, AST and Uric acid.

   * *P*<0.05. [↑](#footnote-ref-0)
