# Supplementary material for: The inverted U-shaped relationship between weight loss percentage and cardiovascular health scores
Source: Eat Weight Disord. 2023 Oct 24;28(1):87. doi: 10.1007/s40519-023-01619-3 (PMC10598164; doi:10.1007/s40519-023-01619-3)
Supplement: Supplementary file 6 — Supplementary file6 (DOCX 14 KB) [file 40519_2023_1619_MOESM6_ESM.docx]

**Supplementary Table 5.** Association between weight loss percentage and CVH scores after excluded subjects with baseline cardiovascular disease and current pregnancy

| Variable | n | Univariate Model | | Multivariate Model | |
| --- | --- | --- | --- | --- | --- |
|  |  | β (95% CI) | *P* value | β (95% CI) | *P* value |
| Total |  |  |  |  |  |
| Per 1 kg decrease | 10268 | 0.17 (0.15~0.2) | <0.001 | 0.17 (0.15~0.2) | <0.001 |
| Percentage degree of weight loss(%) |  |  |  |  |  |
| <0 | 6311 | 0(Ref) |  | 0(Ref) |  |
| 0~5 | 2762 | 2.27 (1.65~2.88) | <0.001 | 2.77 (2.21~3.32) | <0.001 |
| 5.1~10 | 830 | 2.06 (1.07~3.05) | <0.001 | 2.58 (1.69~3.47) | <0.001 |
| 10.1~15 | 251 | 0.87 (-0.87~2.6) | 0.326 | 0.5 (-1.04~2.05) | 0.522 |
| 15.1~20 | 64 | -2.2 (-5.58~1.18) | 0.203 | -2.47 (-5.46~0.52) | 0.106 |
| >20 | 50 | -1.22 (-5.04~2.61) | 0.532 | -1.43 (-4.81~1.95) | 0.407 |
| Normal weight |  |  |  |  |  |
| Per 1 kg decrease | 3305 | -0.19 (-0.24~-0.14) | <0.001 | -0.03 (-0.08~0.02) | 0.206 |
| Percentage degree of weight loss(%) |  |  |  |  |  |
| <0 | 1588 | 0(Ref) |  | 0(Ref) |  |
| 0~5 | 1096 | -0.91 (-1.73~-0.09) | 0.03 | 0.95 (0.19~1.71) | 0.014 |
| 5.1~10 | 422 | -3.9 (-5.05~-2.76) | <0.001 | -0.8 (-1.87~0.26) | 0.139 |
| 10.1~15 | 139 | -5.67 (-7.52~-3.82) | <0.001 | -2.35 (-4.06~-0.64) | 0.007 |
| 15.1~20 | 33 | -7.92 (-11.6~-4.24) | <0.001 | -5.27 (-8.62~-1.93) | 0.002 |
| >20 | 27 | -8.86 (-12.92~-4.8) | <0.001 | -4.97 (-8.67~-1.27) | 0.008 |
| Underweight |  |  |  |  |  |
| Per 1 kg decrease | 173 | -0.29 (-0.61~0.04) | 0.083 | -0.04 (-0.39~0.32) | 0.829 |
| Percentage degree of weight loss(%) |  |  |  |  |  |
| <0 | 55 | 0(Ref) |  | 0(Ref) |  |
| 0~5 | 57 | 2.08 (-3.13~7.3) | 0.435 | 1.41 (-3.77~6.59) | 0.596 |
| 5.1~10 | 31 | 0.22 (-5.98~6.42) | 0.945 | 1.82 (-4.35~7.99) | 0.565 |
| 10.1~15 | 19 | -3.06 (-10.4~4.29) | 0.416 | 0.71 (-6.96~8.38) | 0.856 |
| 15.1~20 | 6 | -3.05 (-14.92~8.82) | 0.615 | 0.45 (-12.11~13) | 0.944 |
| >20 | 5 | -19.31 (-32.2~-6.42) | 0.004 | -13.16 (-26.68~0.35) | 0.058 |
| Overweight / general obesity |  |  |  |  |  |
| Per 1 kg decrease | 3662 | -0.05 (-0.1~0) | 0.034 | 0.07 (0.02~0.11) | 0.004 |
| Percentage degree of weight loss(%) |  |  |  |  |  |
| <0 | 2325 | 0(Ref) |  | 0(Ref) |  |
| 0~5 | 1009 | -0.39 (-1.21~0.43) | 0.352 | 1.04 (0.25~1.83) | 0.01 |
| 5.1~10 | 251 | -1.99 (-3.43~-0.54) | 0.007 | -0.23 (-1.62~1.15) | 0.741 |
| 10.1~15 | 53 | -1.13 (-4.14~1.89) | 0.465 | 0.39 (-2.47~3.25) | 0.79 |
| 15.1~20 | 12 | -1.83 (-8.12~4.46) | 0.568 | -0.77 (-6.72~5.17) | 0.798 |
| >20 | 12 | 2.39 (-3.89~8.68) | 0.456 | 2.88 (-3.05~8.82) | 0.341 |
| Abdominal obesity |  |  |  |  |  |
| Per 1 kg decrease | 266 | -0.44 (-0.69~-0.19) | 0.001 | -0.23 (-0.46~-0.01) | 0.046 |
| Percentage degree of weight loss(%) |  |  |  |  |  |
| <0 | 176 | 0(Ref) |  | 0(Ref) |  |
| 0~5 | 62 | -3.89 (-7.14~-0.64) | 0.02 | -0.63 (-3.62~2.37) | 0.682 |
| 5.1~10 | 0 |  |  |  |  |
| 10.1~15 | 25 | -9.39 (-14.09~-4.69) | <0.001 | -4.1 (-8.51~0.31) | 0.07 |
| 15.1~20 | 3 | -20.85 (-33.64~-8.05) | 0.002 | -17.14 (-28.73~-5.56) | 0.004 |
| >20 | 0 |  |  |  |  |
| Compound ­obesity |  |  |  |  |  |

| Per 1 kg decrease | 2862 | -0.04 (-0.07~0) | 0.028 | 0 (-0.04~0.03) | 0.952 |
| --- | --- | --- | --- | --- | --- |
| Percentage degree of weight loss(%) |  |  |  |  |  |
| <0 | 2167 | 0(Ref) |  | 0(Ref) |  |
| 0~5 | 538 | -1.45 (-2.56~-0.34) | 0.01 | -0.25 (-1.33~0.83) | 0.646 |
| 5.1~10 | 101 | -1.94 (-4.28~0.4) | 0.104 | -1.14 (-3.39~1.11) | 0.323 |
| 10.1~15 | 37 | -4.23 (-8.04~-0.42) | 0.03 | -4.21 (-7.87~-0.56) | 0.024 |
| 15.1~20 | 13 | -8.94 (-15.34~-2.55) | 0.006 | -8.26 (-14.41~-2.11) | 0.009 |
| >20 | 6 | -4.78 (-14.18~4.63) | 0.319 | -4.4 (-13.41~4.62) | 0.339 |

^[[1]](#footnote-0)^

1. CVH cardiovascular health (excluding nicotine exposure component); Multivariate Model was adjusted for age, sex, race, family PIR, the educational attainment of household head, attempts to lose weight in past year, ALT, AST and Uric acid. [↑](#footnote-ref-0)
