# Supplementary material for: The inverted U-shaped relationship between weight loss percentage and cardiovascular health scores
Source: Eat Weight Disord. 2023 Oct 24;28(1):87. doi: 10.1007/s40519-023-01619-3 (PMC10598164; doi:10.1007/s40519-023-01619-3)
Supplement: Supplementary file 7 — Supplementary file7 (DOCX 23 KB) [file 40519_2023_1619_MOESM7_ESM.docx]

**Supplementary Table 6.** Association between weight loss percentage and CVH categories after excluded subjects with baseline cardiovascular disease and current pregnancy.

| Variable | Unadjusted Model | | Adjusted Model | |
| --- | --- | --- | --- | --- |
|  | Moderate versus low CVH | High versus low CVH | Moderate versus low CVH | High versus low CVH |
| Percentage of weight loss, kg | 1.01 (1~1.02)* | 1.04 (1.03~ 1.04)* | 1.01 (1~1.02)* | 1.04 (1.03~ 1.05)* |
| Percentage degree of weight loss(%) |  | | | |
| <0 | 1 (Ref) | 1 (Ref) | 1(Ref) | 1 (Ref) |
| 0~5 | 1.07 (0.87~1.31) | 1.43 (1.16~ 1.77)* | 1.11 (0.9~1.38) | 1.74 (1.38~ 2.18)* |
| 5.1~10 | 1.33 (0.92~1.92) | 1.66 (1.14~ 2.42)* | 1.4 (0.95~2.05) | 2.01 (1.35~ 3.01)* |
| 10.1~15 | 1.08 (0.61~1.92) | 1.13 (0.62~ 2.04) | 0.95 (0.52~1.72) | 0.97 (0.52~ 1.83) |
| 15.1~20 | 0.41 (0.19~0.89)* | 0.44 (0.19~ 1)* | 0.39 (0.17~0.86)* | 0.37 (0.15~ 0.93)* |
| >20 | 1.03 (0.32~3.37) | 0.75 (0.21~ 2.66) | 0.91 (0.27~3.04) | 0.64 (0.17~ 2.41) |

^[[1]](#footnote-0)^

1. CVH cardiovascular health (excluding nicotine exposure component); Adjusted Model was adjusted for age, sex, race, family PIR, the educational attainment of household head, attempts to lose weight in past year, ALT, AST and Uric acid.

   * *P*<0.05. [↑](#footnote-ref-0)
