# Supplementary material for: The inverted U-shaped relationship between weight loss percentage and cardiovascular health scores
Source: Eat Weight Disord. 2023 Oct 24;28(1):87. doi: 10.1007/s40519-023-01619-3 (PMC10598164; doi:10.1007/s40519-023-01619-3)
Supplement: Supplementary file 8 — Supplementary file8 (DOCX 14 KB) [file 40519_2023_1619_MOESM8_ESM.docx]

**Supplementary Table 7.** Association between weight loss percentage and the CVH scores (including nicotine exposure component) across different obesity patterns.

| Variable | n | Univariate Model | | Multivariate Model | |
| --- | --- | --- | --- | --- | --- |
|  |  | β (95% CI) | *P* value | β (95% CI) | *P* value |
| Total |  |  |  |  |  |
| Per 1 kg decrease | 4441 | 0.19 (0.15~0.22) | <0.001 | 0.16 (0.13~0.2) | <0.001 |
| Percentage degree of weight loss(%) |  |  |  |  |  |
| <0 | 2669 | 0(Ref) |  | 0(Ref) |  |
| 0~5 | 1101 | 2.62 (1.74~3.5) | <0.001 | 2.5 (1.66~3.34) | <0.001 |
| 5.1~10 | 431 | 1.3 (0.02~2.57) | 0.046 | 1.32 (0.1~2.54) | 0.034 |
| 10.1~15 | 161 | 1.1 (-0.89~3.1) | 0.278 | 0.88 (-1.02~2.77) | 0.365 |
| 15.1~20 | 45 | -2.46 (-6.15~1.24) | 0.193 | -1.83 (-5.31~1.65) | 0.303 |
| >20 | 34 | 0.33 (-3.92~4.57) | 0.88 | -0.4 (-4.38~3.59) | 0.845 |
| Normal weight |  |  |  |  |  |
| Per 1 kg decrease | 1551 | 0 (-0.06~0.06) | 0.994 | 0.02 (-0.04~0.08) | 0.486 |
| Percentage degree of weight loss(%) |  |  |  |  |  |
| <0 | 735 | 0(Ref) |  | 0(Ref) |  |
| 0~5 | 438 | 0.96 (-0.34~2.26) | 0.148 | 0.99 (-0.27~2.25) | 0.123 |
| 5.1~10 | 234 | -2.37 (-3.99~-0.76) | 0.004 | -1.4 (-2.98~0.18) | 0.083 |
| 10.1~15 | 93 | -2.42 (-4.78~-0.05) | 0.046 | -0.74 (-3.01~1.53) | 0.523 |
| 15.1~20 | 28 | -5.32 (-9.47~-1.18) | 0.012 | -3.29 (-7.2~0.63) | 0.1 |
| >20 | 23 | -4.41 (-8.97~0.15) | 0.058 | -3.14 (-7.45~1.16) | 0.153 |
| Underweight |  |  |  |  |  |
| Per 1 kg decrease | 76 | -0.1 (-0.59~0.39) | 0.692 | 0.03 (-0.53~0.6) | 0.914 |
| Percentage degree of weight loss(%) |  |  |  |  |  |
| <0 | 20 | 0(Ref) |  | 0(Ref) |  |
| 0~5 | 25 | 2.39 (-5.91~10.7) | 0.574 | 4.6 (-4.19~13.4) | 0.309 |
| 5.1~10 | 14 | 4.25 (-5.39~13.9) | 0.39 | 4.88 (-5.85~15.6) | 0.376 |
| 10.1~15 | 13 | -0.66 (-10.52~9.21) | 0.897 | 1.83 (-8.87~12.53) | 0.738 |
| 15.1~20 | 2 | 6.53 (-14~27.06) | 0.535 | 13.83 (-15.07~42.73) | 0.352 |
| >20 | 2 | -15.66 (-36.19~4.88) | 0.14 | -8.52 (-31.01~13.97) | 0.461 |
| Overweight / general obesity |  |  |  |  |  |
| Per 1 kg decrease | 1575 | 0.12 (0.05~0.19) | <0.001 | 0.11 (0.04~0.17) | 0.002 |
| Percentage degree of weight loss(%) |  |  |  |  |  |
| <0 | 997 | 0(Ref) |  | 0(Ref) |  |
| 0~5 | 412 | 1.12 (-0.15~2.39) | 0.083 | 1.39 (0.15~2.62) | 0.028 |
| 5.1~10 | 114 | -0.72 (-2.87~1.42) | 0.507 | 0.17 (-1.9~2.23) | 0.874 |
| 10.1~15 | 37 | -0.51 (-4.14~3.12) | 0.782 | 0.44 (-3.03~3.91) | 0.804 |
| 15.1~20 | 7 | -1.97 (-10.19~6.24) | 0.638 | 0.79 (-7.04~8.62) | 0.844 |
| >20 | 8 | -0.59 (-8.28~7.1) | 0.881 | 0.46 (-6.87~7.8) | 0.902 |
| Abdominal obesity |  |  |  |  |  |
| Per 1 kg decrease | 109 | -0.12 (-0.46~0.22) | 0.485 | -0.13 (-0.47~0.2) | 0.433 |
| Percentage degree of weight loss(%) |  |  |  |  |  |
| <0 | 70 | 0(Ref) |  | 0(Ref) |  |
| 0~5 | 23 | 0.04 (-5.47~5.55) | 0.988 | 0.95 (-4.18~6.08) | 0.716 |
| 5.1~10 | 0 |  |  |  |  |
| 10.1~15 | 14 | -5.54 (-12.25~1.18) | 0.109 | -1.65 (-8.15~4.85) | 0.62 |
| 15.1~20 | 2 | -25.94 (-42.39~-9.49) | 0.003 | -31.49 (-47.98~-14.99) | <0.001 |
| >20 | 0 |  |  |  |  |
| Compound ­obesity |  |  |  |  |  |

| Per 1 kg decrease | 1130 | 0.06 (0~0.11) | 0.034 | 0.03 (-0.02~0.09) | 0.225 |
| --- | --- | --- | --- | --- | --- |
| Percentage degree of weight loss(%) |  |  |  |  |  |
| <0 | 847 | 0(Ref) |  | 0(Ref) |  |
| 0~5 | 203 | 0.12 (-1.63~1.88) | 0.889 | -0.21 (-1.94~1.52) | 0.811 |
| 5.1~10 | 55 | -2.23 (-5.36~0.89) | 0.161 | -0.94 (-3.95~2.08) | 0.543 |
| 10.1~15 | 16 | -0.96 (-6.62~4.7) | 0.74 | -0.62 (-6.07~4.83) | 0.824 |
| 15.1~20 | 8 | -11.15 (-19.12~-3.19) | 0.006 | -9.35 (-17.03~-1.67) | 0.017 |
| >20 | 1 | 0.25 (-22.19~22.7) | 0.982 | -2.62 (-24.13~18.89) | 0.811 |

^[[1]](#footnote-0)^

1. CVH cardiovascular health (including nicotine exposure component); Multivariate Model was adjusted for age, sex, race, family PIR, the educational attainment of household head, attempts to lose weight in past year, ALT, AST and Uric acid. [↑](#footnote-ref-0)
