# Supplementary material for: The inverted U-shaped relationship between weight loss percentage and cardiovascular health scores
Source: Eat Weight Disord. 2023 Oct 24;28(1):87. doi: 10.1007/s40519-023-01619-3 (PMC10598164; doi:10.1007/s40519-023-01619-3)
Supplement: Supplementary file 9 — Supplementary file9 (DOCX 19 KB) [file 40519_2023_1619_MOESM9_ESM.docx]

**Supplementary Table 8.** Association between weight loss percentage and CVH categories (including nicotine exposure component)

| Variable | Unadjusted Model | | Adjusted Model | |
| --- | --- | --- | --- | --- |
|  | Moderate versus low CVH | High versus low CVH | Moderate versus low CVH | High versus low CVH |
| Percentage of weight loss, kg | 1.03 (1.02~1.03)* | 1.05 (1.03~ 1.06)* | 1.02 (1.01~1.03)* | 1.04 (1.03~ 1.06)* |
| Percentage degree of weight loss(%) |  | | | |
| <0 | 1 (Ref) | 1 (Ref) | 1(Ref) | 1 (Ref) |
| 0~5 | 1.45 (1.13~1.87)* | 1.96 (1.46~ 2.62)* | 1.37 (1.06~1.78)* | 1.96 (1.43~ 2.68)* |
| 5.1~10 | 1.36 (0.95~1.94) | 1.35 (0.88~ 2.08) | 1.21 (0.84~1.75) | 1.33 (0.84~ 2.1) |
| 10.1~15 | 1.69 (0.92~3.08) | 1.33 (0.64~ 2.79) | 1.35 (0.73~2.51) | 1.21 (0.56~ 2.62) |
| 15.1~20 | 0.64 (0.29~1.4) | 0.42 (0.13~ 1.41) | 0.58 (0.26~1.29) | 0.46 (0.13~ 1.66) |
| >20 | 4.51 (0.61~33.25) | 3.37 (0.37~ 30.28) | 3.5 (0.47~26.03) | 2.63 (0.28~ 24.57) |

^[[1]](#footnote-0)^

1. CVH cardiovascular health (including nicotine exposure component); Adjusted Model was adjusted for age, sex, race, family PIR, the educational attainment of household head, attempts to lose weight in past year, ALT, AST and Uric acid.

   * *P*<0.05. [↑](#footnote-ref-0)
