# Supplementary material for: The inverted U-shaped relationship between weight loss percentage and cardiovascular health scores
Source: Eat Weight Disord. 2023 Oct 24;28(1):87. doi: 10.1007/s40519-023-01619-3 (PMC10598164; doi:10.1007/s40519-023-01619-3)
Supplement: Supplementary file 10 — Supplementary file10 (DOCX 52 KB) [file 40519_2023_1619_MOESM10_ESM.docx]

**Supplementary Table 9.** Reported Weight Loss Strategies by Percentage degree of weight loss

| Variable | Total (n = 5421) | <0% (n = 3842) | 0-5% (n = 1196) | 5.1-10% (n = 304) | 10.1-15% (n = 51) | 15.1-20% (n = 15) | >20%(n = 13) | *P* value |
| --- | --- | --- | --- | --- | --- | --- | --- | --- |
| Weight loss strategy |  |  |  |  |  |  |  |  |
| Ate less to lose weights^*^ | 3154 (58.2) | 2263 (58.9) | 679 (56.8) | 170 (55.9) | 24 (47.1) | 10 (66.7) | 8 (61.5) | 0.355 |
| Switched to foods with lower calories | 1827 (33.7) | 1314 (34.2) | 405 (33.9) | 86 (28.3) | 12 (23.5) | 4 (26.7) | 6 (46.2) | 0.14 |
| Ate less fat to lose weight^*^ | 1841 (34.0) | 1293 (33.7) | 420 (35.1) | 108 (35.5) | 11 (21.6) | 6 (40) | 3 (23.1) | 0.319 |
| Exercised to lose weights^*^ | 4312 (79.5) | 3056 (79.5) | 959 (80.2) | 239 (78.6) | 36 (70.6) | 13 (86.7) | 9 (69.2) | 0.49 |
| Skipped meals | 874 (16.1) | 652 (17) | 163 (13.6) | 41 (13.5) | 10 (19.6) | 6 (40) | 2 (15.4) | 0.009 |
| Ate diet foods or products | 519 ( 9.6) | 401 (10.4) | 84 (7) | 28 (9.2) | 4 (7.8) | 1 (6.7) | 1 (7.7) | 0.02 |
| Used a liquid diet formula | 277 ( 5.1) | 195 (5.1) | 59 (4.9) | 18 (5.9) | 3 (5.9) | 1 (6.7) | 1 (7.7) | 0.769 |
| Joined a weight loss program^*^ | 274 ( 5.1) | 214 (5.6) | 49 (4.1) | 10 (3.3) | 1 (2) | 0 (0) | 0 (0) | 0.205 |
| Took prescription diet pills | 103 ( 1.9) | 86 (2.2) | 10 (0.8) | 3 (1) | 3 (5.9) | 0 (0) | 1 (7.7) | 0.002 |
| Took nonprescription supplement to lose weight | 333 ( 6.1) | 249 (6.5) | 61 (5.1) | 17 (5.6) | 3 (5.9) | 2 (13.3) | 1 (7.7) | 0.305 |
| Took laxatives or vomited | 55 ( 1.0) | 38 (1) | 13 (1.1) | 2 (0.7) | 1 (2) | 1 (6.7) | 0 (0) | 0.266 |
| Drank a lot of water | 2587 (47.7) | 1895 (49.3) | 508 (42.5) | 147 (48.4) | 22 (43.1) | 10 (66.7) | 5 (38.5) | 0.001 |
| Followed a special diet^*^ | 312 ( 5.8) | 233 (6.1) | 58 (4.8) | 16 (5.3) | 3 (5.9) | 1 (6.7) | 1 (7.7) | 0.561 |
| Ate fewer carbohydrates | 1498 (27.6) | 1053 (27.4) | 353 (29.5) | 74 (24.3) | 13 (25.5) | 2 (13.3) | 3 (23.1) | 0.375 |
| Started to smoke or began to smoke again | 40 ( 0.7) | 23 (0.6) | 7 (0.6) | 5 (1.6) | 5 (9.8) | 0 (0) | 0 (0) | < 0.001 |
| Ate more fruits, vegetables, salads^*^ | 2337 (43.1) | 1662 (43.3) | 515 (43.1) | 130 (42.8) | 17 (33.3) | 5 (33.3) | 8 (61.5) | 0.49 |
| Changed eating habits^*^ | 1647 (30.4) | 1189 (30.9) | 344 (28.8) | 93 (30.6) | 13 (25.5) | 5 (33.3) | 3 (23.1) | 0.702 |
| Ate less sugar, candy, sweets^*^ | 1682 (31.0) | 1198 (31.2) | 361 (30.2) | 106 (34.9) | 10 (19.6) | 4 (26.7) | 3 (23.1) | 0.317 |
| Other | 47 ( 0.9) | 30 (0.8) | 8 (0.7) | 5 (1.6) | 0 (0) | 2 (13.3) | 2 (15.4) | < 0.001 |
| Minutes of activity per week | 240.0 (120.0, 480.0) | 240.0 (120.0, 490.0) | 240.0 (120.0, 480.0) | 240.0 (120.0, 496.2) | 240.0 (110.0, 420.0) | 225.0 (112.5, 705.0) | 180.0 (160.0, 240.0) | 0.834 |
| average daily intake of kcal/kg | 43.6 (32.1, 56.8) | 42.6 (31.3, 55.7) | 46.4 (35.3, 59.6) | 48.2 (35.6, 60.5) | 37.9 (26.7, 47.2) | 38.7 (31.0, 69.1) | 40.8 (29.0, 65.4) | < 0.001 |
| sleep duration | 7.1 ± 1.3 | 7.1 ± 1.4 | 7.1 ± 1.3 | 7.2 ± 1.4 | 6.5 ± 1.6 | 6.6 ± 1.7 | 7.4 ± 1.7 | 0.01 |

^[[1]](#footnote-0)^

1. Individuals who reported a weight loss attempt over the past 12 months were asked to provide details about the weight loss strategies they used. Data above represent responses only for those who provided these details(n = 5421).

   ^*^Indicates strategies that most closely align with evidence-­based behavioral recommendations for weight loss. [↑](#footnote-ref-0)
